# A single center cohort of 40 severe COVID-19 patients who were treated with convalescent plasma

**DOI:** 10.3906/sag-2009-77

**Published:** 2020-12-17

**Authors:** Aliihsan GEMİCİ, Hülya BİLGEN, Cem ERDOĞAN, Abdullah KANSU, Oktay OLMUŞÇELİK, Hüseyin Saffet BEKÖZ, Rümeysa DİNLEYİCİ, Ali MERT, Ömür Gökmen SEVİNDİK

**Affiliations:** 1 Department of Hematology, School of Medicine, İstanbul Medipol University, İstanbul Turkey; 2 Department Transfusion and Apheresis Medicine, School of Medicine, İstanbul Medipol University, İstanbul Turkey; 3 Department of Anesthesiology and Reanimation, School of Medicine, İstanbul Medipol University, İstanbul Turkey; 4 Department of Pulmonology, School of Medicine, İstanbul MedipolUniversity, İstanbul Turkey; 5 Department of Internal Medicine, School of Medicine, İstanbul Medipol University, İstanbul Turkey; 6 Department of Clinical Pharmacy, School of Medicine, İstanbul Medipol University, İstanbul Turkey

**Keywords:** Convalescent plasma, COVID-19, viral infections

## Abstract

**Background/aim:**

A SARS-Cov2 infection which was first arised from Wuhan in December 2019 and named as COVID-19. Still there lacks either a specific treatment or a vaccine to treat COVID-19. Convalescent plasma (CP) was previously used successfully to treat SARS-CoV-1 and MERS infections. Health authority in Turkey has published a guideline to integrate this promising option in the treatment process of patients who are prone to high risk of developing severe COVID 19.

**Materials and Methods:**

Forty consecutive patients who had received CP at our center were included in the study. Demographics, COVID-19 specific parameters, biomarkers to detect the severity of COVID-19 infection and outcome variables were collected retrospectively. The correlation between outcome variables and the independent predictors of the outcome were reported.

**Results:**

Median age of the patients was 57.5 and 72.5% were male. At least one COVID-19 PCR test was confirmed to be positive in 75% of patients. Remaining 25% had a Chest-CT which was reported to be compatible with an ongoing COVID-19. All patients (100%) were classified as having severe COVID-19 infection. Over a half of the patients harbored an oxygen saturation of less than 90 despite of a continuous 5 L/min support of O2. 82.5% of the patients had a need for mechanical ventilation and 45.5% had a need for invasive mechanical ventilation. Nine out of 10 patients who have received CP outside ICU have totally recovered from COVID-19 at a median of 9 days, and a half of the patients who needed invasive mechanical ventilation were successfully free of mechanical ventilation support and managed to recover from COVID-19.

**Conclusion:**

According to the results of this study, CP is an efficient conjunct to conventional therapy against COVID-19 with a favorable safety profile.

## 1. Introduction

Since December 2019, a pneumonia associated with severe acute respiratory syndrome coronavirus 2 (SARS-CoV-2), named as coronavirus disease 2019 (COVID-19) by World Health Organization (WHO), emerged in Wuhan, China 1World Health Organization (2020). Coronavirus disease (COVID-2019) press briefings [online]. Website https://www.who.int/emergencies/diseases/novel-coronavirus-2019/media-resources/press-briefings [accessed 11/03/2020].
. The epidemic spread rapidly worldwide within 3 months and was characterized as a pandemic by the WHO on March 11, 2020. To date, there are no proven option for prophylaxis for those who have been exposed to SARS-CoV-2, nor therapy for those who develop COVID-19. Therapeutic strategy for COVID-19 is largely supportive. Since the effective vaccine and specific antiviral medicines are unavailable, it is an urgent need to look for an alternative strategy to treat COVID-19, especially among severe patients 2Turkish Republic Ministry of Health (2020). COVID-19 Bilgilendirme Sayfası [online]. Website https://covid19.saglik.gov.tr/ [accessed 10/04/2020].
.

Immune (i.e. “convalescent”) plasma refers to plasma that is collected from individuals, following resolution of infection and development of antibodies [1]. There are numerous examples, where CP has been used successfully as postexposure prophylaxis and/or treatment of infectious diseases, including other outbreaks of coronaviruses (e.g., SARS-CoV-1, Middle East respiratory syndrome [MERS]) [2]. However, the efficacy of CP in critically ill patients with SARS-CoV-2 infection remains unclear [3–6]. Health authority in Turkey, has rapidly established a scientific committee to standardize the treatment of COVID-19. As novel data emerged showing a potential benefit of immune plasma therapy, the scientific committee and health authority has published a guideline to integrate this promising approach in the treatment process of patients who are prone to high risk of developing severe COVID-19. In line with the directives of Ministry of Health, we have started to use immune plasma therapy institutionally from 7th of April 20202.

Here we share our experience regarding the immune plasma therapy and aimed to document the efficacy and safety of this treatment approach.

## 2. Methods

### 2.1. Patient selection and ethics

Medipol Hospital Complexes have been converted to a dedicated pandemic hospital as soon as the first cases were diagnosed with COVID-19 in Turkey. We have hospitalized 865 patients as of 28th of April. We have institutionally followed the guidelines of Ministry of Health. With the advent of immune plasma specific guidelines, we have initiated a dedicated service to collect and provide immune plasma from healthy donors who has previously recovered from COVID-19. Criteria to detect eligible patient were as follows:

• To harbor CT signs which can be attributable to a COVID-19 and at least 50% deterioration of these signs in 24–48 h,

• A minute respiratory rate over 30,

• A PaO _2_ /FiO _2_ level of less than 300 mm Hg,

• An oxygen saturation of less than 90% despite of at least 5 L/min nasal oxygen support,

• A PaO _2_ of less than 90% despite of at least 5 L/min nasal oxygen support,

• Need for mechanical ventilation,

• At least two points increase in SOFA score,

• Need for vasopressor support,

• To harbor at least one poor prognostic factor and patients who are supposed to deteriorate rapidly (severe lymphopenia, severe CRP, sedimentation rate, ferritin, LDH, D-dimer elevation).

Approval of an institutional committee consisting of an infectious disease professional, pulmonology professional and intensivist were required, and a technical consultation of hematologist and apheresis unit was made before selecting the potential patient.

With regard to the emergent need for any potential treatment of COVID-19, both Turkish Ministry of Health and FDA has approved the use of CP against COVID-19 without a need for measuring the neutralizing antibody titers. That is why we have infused CP products without obtaining or waiting for the results of neutralizing antibody titers.

All patients or a first degree relative of the patient has approved a written informed consent detailing the procedure and potential side effects.

### 2.2. Donor selection

Healthy donors were also detected in line with the directives of health authority.

The criteria to define a potential donor were as follows:

• To have at least one positive laboratory measure which proves the presence of previous COVID-19.

• If the potential donor was hospitalized with a diagnosis of COVID-19, at least two consecutive negative laboratory results indicating the absence or disappearance and clearance of COVID-19 with a symptom free period of at least 14 days.

• If the potential donor was not hospitalized, and followed up at home, with a diagnosis of COVID-19, at least one negative laboratory results indicating the absence or disappearance and clearance of COVID-19 with a symptom free period of at least 28 days.

• To fulfill the conditions of national blood component donor selection criteria despite of previous viral infection in last 28 days.

The donors needed to be seronegative for anti-HBV, HCV and HIV.

200–600 mL CP was collected by apheresis.

### 2.3. Safety and therapeutic outcome evaluation

We retrospectively collected demographic and clinical data among patients who had at least one of these aforementioned criteria and supported with at least one infusion of immune plasma blood product.

Adverse events and serious adverse events associated with CP transfusion were assessed by the treating clinician.

The primary outcome was the improvement in symptoms and chest CT if available in the following days after indicated intervention. Blood and swab samples were obtained to measure serum anti-SARS-CoV-2 IgM and IgG titers and throat SARS-CoV-2 nucleic acid, respectively.

### 2.4. Statistical analysis

Continous variables were expressed as median (range) and categorical variables were expressed as number (percent). Descriptive statistics were used as indicated. Comparisons between categorical variables were measured using chi‐square test. Mann–Whitney U test was used to compare the distribution of continuous variables between 2 independent groups.

All statistical tests were two sided, and analyses were performed with SAS 9.4 (SAS Institute Inc., Cary, NC, USA). A P-value less than 0.05 was accepted as statistically significant.

## 3. Results

Median age of the 40 patients who received at least one CP infusion was 57.5. Seventy-two and one half percent of the patients were male and 57.5% of the patients were grouped as having an “A” blood type. Seventy-five percent of the patients had a positive COVID-19 PCR test and the remaining patients harbored typical CT findings attributable to COVID-19. Median time from the diagnosis to the infusion of the first CP was 5 days (Table 1). Near all patients complained of dyspnea, and 77.5% of the patients had a PaO _2_ /FiO _2_ level less than 300. Forty-five percent of the patients needed invasive mechanical ventilation, and 25% required additional vasopressor support.

**Table 1:** Patient demographics.

	All patients	Non-ICU patients(n = 10)	ICU patients(n = 30)	P Value
Age	57.5 (35–82)	51 (44–82)	60 (35–73)	0.230
Sex (M/F %)	72.5 / 27.5	66.7 / 33.3	74.2 / 25.8	0.686
Blood typing (n)				
A Rh + / A Rh -	19 / 4	2 / 1	16 / 3	0.096 *
B Rh + / B Rh -	5 / 2	2 / 1	3 / 1	
AB Rh + / AB Rh -	3 / 1	3 / 0	1 / 1	
0 Rh + / 0 Rh -	5 / 1	1 / 1	4 / 0	
COVID 19 PCR positivity - chest CT positivity	75–25	100–0	67.7–33.3	0.081
Interval between diagnosis and first plasma infusion (days)	5 (1–17)	9 (1–16)	4 (2–17)	0.253

* Comparison was made between patients with “A” blood type vs. other blood types among ICU vs. non-ICU groups.

When compared with patients who have received the first CP in intensive care unit (ICU), patients who have received their CP outside the ICU had a favorable pulmonary profile (Table 2) and they also had a more favorable biomarker profile (Table 3).

**Table 2:** Symptom burden.

	Allpatients	Non-ICU patients (n = 10)	ICU patients(n = 30)	P value
Dyspnea (%)	95	88.9	96.8	0.465
Tachypnea (%)	85	55.6	93.5	0.004
PaO _2_ /FiO _2_ < 300 mmHg (%)	77.5	55.6	83.9	0.012
SaO2< 90 mmHg (despite of a continuous 5 L/min support of O2) (%)	75	66.7	77.4	0.005
PaO _2_ < 70 mmHg (despite of a continuous 5 L/min support of O2) (%)	57.5	33.3	64.5	0.008
Need for mechanical ventilation (%)	82.5	22.2	100	<0.001
Need for invasive mechanical ventilation (%)	45.5	0	48.4	<0.001
Need for vasopressors (%)	25	0	32.3	0.006

**Table 3:** Presence of poor prognostic biomarkers.

	Allpatients	Non-ICUpatients	ICUpatients	P value
High ferritin (%)	52.5	33.3	58.1	0.014
High CRP (%)	92.5	100	90.3	0.453
High D-dimer (%)	87.5	55.6	96.8	0.002
Presence of lymphopenia (%)	92.5	88.4	93.5	0.354

Nine out of the 10 patients who have received the first infusion outside the ICU have succeeded to recover from COVID-19 and 1 patient was transferred to ICU despite having CP infusion and unfortunately lost due to worsening ARDS. A total of 15 patients who have received the CP infusion in ICU were succeeded to be free of either noninvasive (n = 11, 73.3%) or invasive mechanical (n = 4, 26.7%) ventilation and discharged successfully (Figure).

**Figure:**
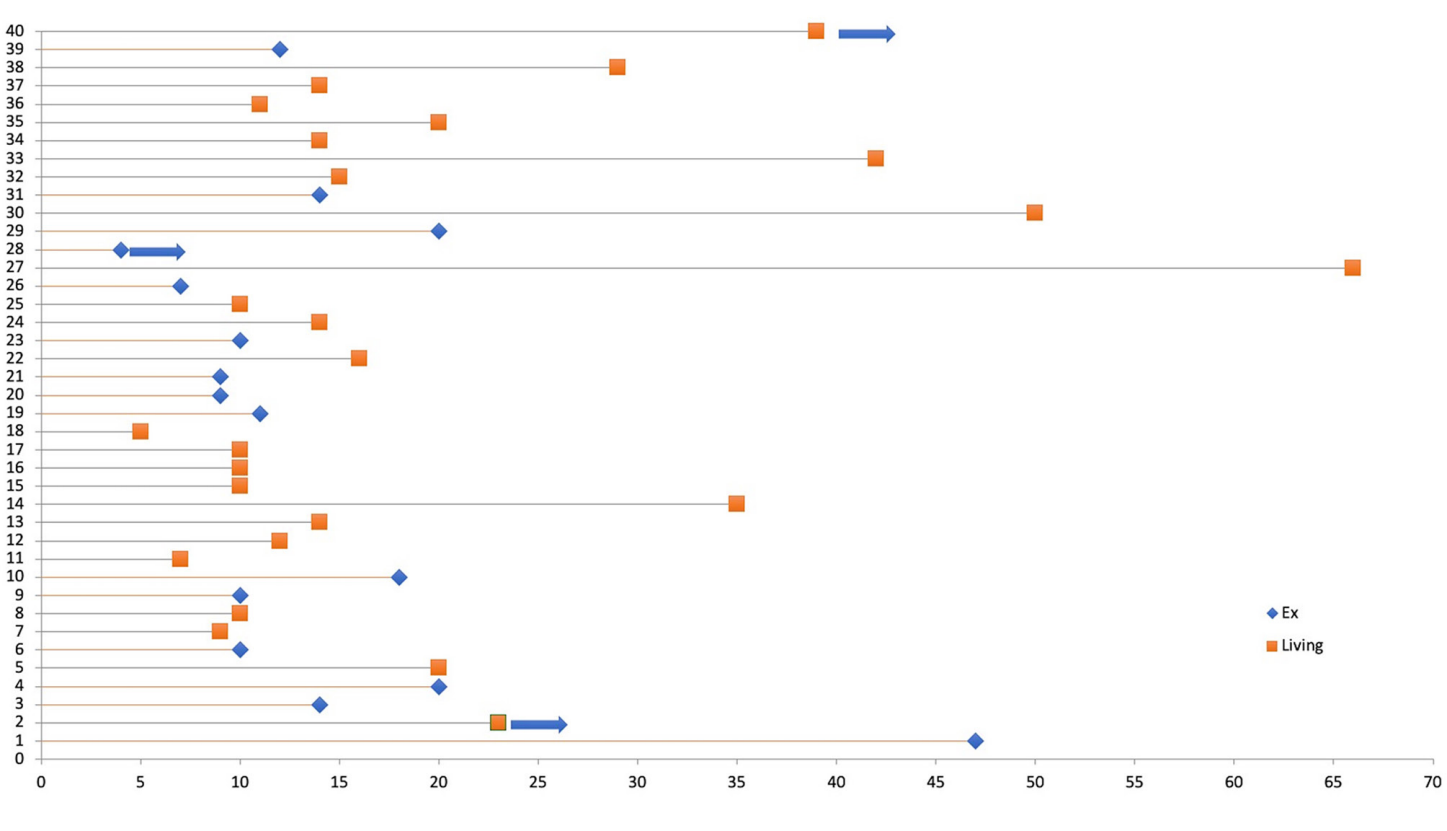
Time to death or discharge from the first infusion of CP.

Patients have received a median of 2 (1–3) CP infusions. Twenty-four (60%) out of the 40 patients have needed a repeated infusion of the CP at the discretion of the following physician.

All of patients have received additional antiviral therapy (favipiravir) and 35% of the patients have received additional anti IL-6 therapy, tocilizumab before the first CP infusion.

## 4. Discussion

There exists limited data of the use of CP therapy in viral infections which can cause ARDS. Previous experience of this immune therapy has shown promising efficacy with a favorable safety profile [7–9]. With this regard, as currently there exists no specific treatment option of COVID 19, national health authority has accepted CP therapy as a valid option and developed a national guideline to optimize the use of aforementioned therapeutic approach.

We tried to document the efficacy and safety of immune plasma therapy in our 40 consecutive patients treated at our tertiary center which was a dedicated pandemic hospital. We have hospitalized 865 patients beginning from 11th of March to 28th of April. Forty severe COVID-19 patients have received CP therapy, in both intensive care unit and nonintensive care wards.

According to the results obtained from our study, 15 patients who received immune plasma therapy have managed to be free of mechanical ventilation in a median of 14 days, even they had a prolonged time from initiation of first symptoms to immune plasma infusion when compared with the patients who had their infusion in ICU. This ratio of getting free of invasive mechanical ventilation was slightly higher than the rates reported in previous trials [7, 8,10].

All patients who were classified as having severe COVID 19 and did not transferred to ICU, have managed to be cured of disease. A half of the patients who were followed in ICU care have managed to be extubated after the application of immune plasma therapy.

None of the patients who were included in the study has experienced any grade 2 or more toxicity which could be related to CP infusion. One of the patients had an episode of fever after the first infusion of CP. No TRALI (transfusion-related acute lung injury) or severe allergic reactions were documented. Recently an analysis of 5000 patients who received CP has also confirmed infusion of CP as a safe therapeutic option [9].

In this current pandemic, there are reports that CP has been used in China to treat patients with COVID- 19. In a pilot study of 10 patients with severe COVID-19, the investigators collected CP with neutralizing antibody titers at or exceeding a 1:640 dilution [8]. Transfusion of CP resulted in no serious adverse effect in the recipients. All 10 patients had improvement in symptoms (e.g., fever, cough, shortness of breath and chest pain) within 1–3 days of transfusion; they also demonstrated radiological improvement in pulmonary lesions. In 7 RNA-emic patients, transfusion of CP was temporally associated with undetectable viral loads. Further, screening of 39 of 40 (97.5%) of recovered COVID-19 patients displayed neutralizing antibody titers ≥160. A case series of 5 critically ill patients in China also reported improvement in clinical status following transfusion with CP (SARS-CoV-2 IgG titers >1000) as evidenced by weaning off mechanical ventilation, reduction in viral loads, improved oxygenation and clinical stabilization [7].

Our results were also comparable with a recent publication of Altuntas et.al, in which they have concluded that, CP therapy seems to be effective for a better course of COVID-19 in severe and critically ill patients in a Turkish patient cohort [11].

Retrospective nature of the study and unavailable neutralizing antibody titers of the donors were the main limitations of our study. Despite of these limitations we appreciate the effort of health authority in Turkey to promote the use of immune plasma treatment even in lack of currently available antibody titers, as also indicated in FDA approval of this approach.

One other limitation of this study was its nonrandomized nature and unstandardized protocol. Unfortunately, as there exists no specific treatment of COVID-19, near all patients have received other concomitant therapies, like; hydroxychloroquine, azithromycin, favipiravir, tocilizumab, anakinra.

We conclude that immune plasma infusion is an efficient and safe adjunct treatment in COVID-19. And best outcome should be achieved when applied prior to the need for ICU care.

## Disclaimer/Conflict of interest

All authors declare no competing financial disclosure. All authors declare no competing conflict of interest.
